# BLISTER FLUID IMMUNOFLUORESCENCE IN A CASE OF PEMPHIGUS VULGARIS

**DOI:** 10.4103/0019-5154.62755

**Published:** 2010

**Authors:** C Shanmugasekar, V R Ram Ganesh, Alamelu Jayaraman, C R Srinivas

**Affiliations:** *From the Department of Dermatology, PSG Hospitals, Coimbatore, India.*; 1 *From the Department of Pathology, PSG Hospitals, Coimbatore, India.*

**Keywords:** *Blister*, *immunofluorescence*, *pemphigus*

## Abstract

Indirect immunofluorescence with serum is used in the diagnosis of pemphigus. We report a case in whom blister fluid was used as the specimen for indirect immunofluorecscence.

## Introduction

Pemphigus vulgaris is an autoimmune blistering disease characterized histologically by suprabasal blister and immunopathologically by *in vivo* bound and circulating antibodies directed against the desmogleins. Immunofluorescence is one of the diagnostic tests done in cases of pemphigus. In 1941, Coons *et al.* developed the technique of immunofluorescence. In 1964, Beutner and Jordon used the indirect immunofluorescence (IIF) technique to demonstrate antibodies in the sera of pemphigus patients.[1] We report a case of pemphigus in whom indirect immunofluorescence (IIF) was done with blister fluid.

## Case Report

**Figure 1:**
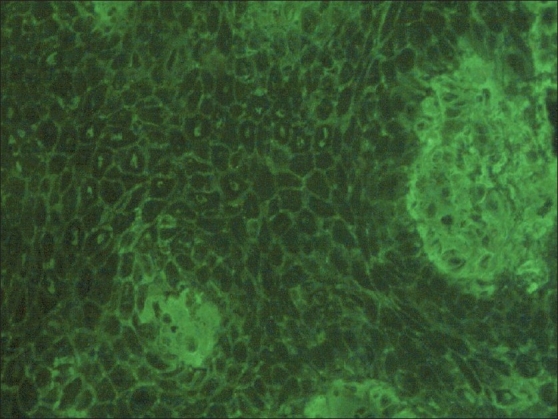
Blister fluid immunofluorescence IgG 1:10 dilution

**Figure 2:**
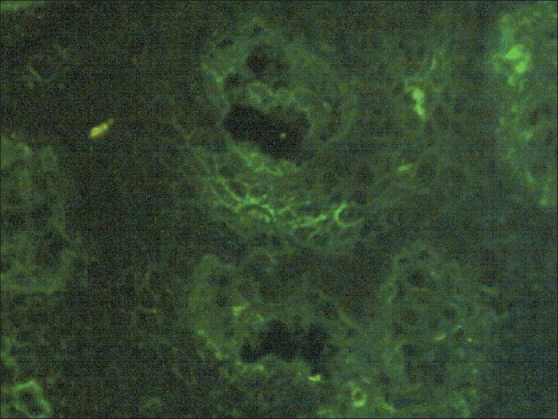
Blister fluid immunofluorescence C3 1:10 dilution

A 30-year-old female a known case of pemphigus was biopsied for histopathology and direct immunofluorescence (DIF). Blister fluid along with serum was taken for indirect immunofluorescence (IIF). DIF showed strongly positive IgG deposits. Serial dilutions of blister fluid using phosphate buffered saline (PBS) were incubated with cryostat sections of normal skin (substrate). Subsequently sections were incubated with FITC-labeled anti-human IgG for 30 min at 37°C. Slides were washed with PBS and mounted in buffered glycerol. Fluorescent microscopy revealed moderately strong ICS deposits of IgG (1:10, 1: 80) and focal weak ICS deposits of C3 (1: 10, 1:80) [Figures 1 and 2]. IIF with serum showed similar results.

## Discussion

Immunofluorescence is nowadays commonly used in the diagnosis of vesicobullous disorders. Direct immunofluorescence requires perilesional skin, whereas serum is used for indirect immunofluorescence. Blisters are formed as a result of a breakdown of tissue integrity and fluid accumulation in a specific compartment of the skin. In pemphigus, simple binding of the antibody to the ectodomain of the antigen (desmoglein 3) triggers blister formation, perhaps by impairing the function of the molecule.[2] Blister fluid has been qualified as a “filtrate” of serum and also contains local products of cell injury and inflammation. There are number of case reports of blister fluid used in diagnosis of subepidermal disorders. Daneshpazhooh *et al.*[3] compared the antibody titers of blister fluid and serum in patients with subepidermal immunobullous diseases and concluded that IIF sensitivity on blister fluid is no more than that on serum, but the performance of this test on blister fluid in addition to serum may reduce the number of false negative results of IIF found using either of these two substrates alone. Zhou *et al.*[4] compared the basement membrane zone autoantibodies detected in blister fluid and serum for the diagnosis of subepidermal immunobullous diseases and concluded that blister fluid can be used as an alternative to serum for indirect IMF in subepidermal immunobullous diseases. Baroni *et al.*[5] had described the cytokine pattern in blister fluid and sera of patients with pemphigus using the immunoenzymatic assay. There are no reports of blister fluid being used as a specimen for indirect immunofluorescence in pemphigus. We report this case because blister fluid can be easily obtained without any trauma and can be used as an alternative for serum in IIF and may also improve the sensitivity of routine indirect immunofluorescence.
